# Sec-O-Glucosylhamaudol Inhibits RANKL-Induced Osteoclastogenesis by Repressing 5-LO and AKT/GSK3β Signaling

**DOI:** 10.3389/fimmu.2022.880988

**Published:** 2022-04-26

**Authors:** Jinjin Cao, Ming-Xue Zhou, Xinyan Chen, Menglu Sun, Congmin Wei, Qisheng Peng, Zhou Cheng, Wanchun Sun, Hongbing Wang

**Affiliations:** ^1^Putuo People’s Hospital, School of Life Sciences and Technology, Tongji University, Shanghai, China; ^2^Department of Neurology, Ruikang Hospital of Guangxi Traditional Chinese Medicine (TCM) University, Nanning, China; ^3^Key Laboratory of Zoonoses Research, Ministry of Education, Institute of Zoonosis, Jilin University, Changchun, China

**Keywords:** Sec-O-glucosylhamaudol, osteoclasts, NFATc1, GSK3β, 5-lipoxygenase, AKT

## Abstract

Sec-O-glucosylhamaudol (SOG), an active flavonoid compound derived from the root of *Saposhnikovia divaricata* (Turcz. ex Ledeb.) Schischk., exhibits analgesic, anti-inflammatory, and high 5-lipoxygenase (5-LO) inhibitory effects. However, its effect on osteoclastogenesis was unclear. We demonstrated that SOG markedly attenuated RANKL-induced osteoclast formation, F-actin ring formation, and mineral resorption by reducing the induction of key transcription factors NFATc1, c-Fos, and their target genes such as *TRAP*, *CTSK*, and *DC-STAMP* during osteoclastogenesis. Western blotting showed that SOG significantly inhibited the phosphorylation of AKT and GSK3β at the middle–late stage of osteoclastogenesis without altering calcineurin catalytic subunit protein phosphatase-2β-Aα expression. Moreover, GSK3β inhibitor SB415286 partially reversed SOG-induced inhibition of osteoclastogenesis, suggesting that SOG inhibits RANKL-induced osteoclastogenesis by activating GSK3β, at least in part. 5-LO gene silencing by small interfering RNA in mouse bone marrow macrophages markedly reduced RANKL-induced osteoclastogenesis by inhibiting NFATc1. However, it did not affect the phosphorylation of AKT or GSK3β, indicating that SOG exerts its inhibitory effects on osteoclastogenesis by suppressing both the independent 5-LO pathway and AKT-mediated GSK3β inactivation. In support of this, SOG significantly improved bone destruction in a lipopolysaccharide-induced mouse model of bone loss. Taken together, these results suggest a potential therapeutic effect for SOG on osteoclast-related bone lysis disease.

## Introduction

The dynamic process of bone remodeling is regulated by the balance between osteoblast-mediated bone formation and osteoclast-mediated bone resorption (1). Excessive bone resorption by hyperosteoclastogenesis can lead to bone loss, resulting in osteoporosis, rheumatoid arthritis, periodontitis, Paget’s disease, and bone tumors (2). Previous studies suggested that inflammation and oxidative stress promote osteoclast differentiation, causing osteolysis (3, 4). Therefore, the inhibition of excessive osteoclast formation and bone resorption activity has been regarded as an important therapeutic strategy for bone lysis diseases.

Macrophage colony-stimulating factor (M-CSF) and receptor activator of nuclear factor-κB ligand (RANKL) are important cytokines involved in osteoclast differentiation (5, 6). RANKL binds to its receptor, RANK, and rapidly activates nuclear factor (NF)-κB, mitogen-activated protein kinase (MAPK), and c-Fos signaling, which first induce the activation of nuclear factor of activated T cells, cytoplasmic (NFATc)1, followed by its auto-amplification (7, 8). Coordinating with RANKL signaling, immunoreceptor tyrosine-based activation motif signaling regulates the transcriptional activity of *NFATc1* (9).

**Figure 1:**
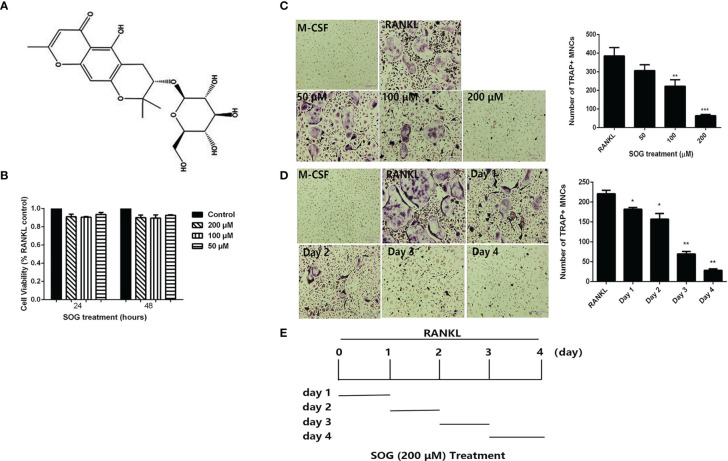
SOG inhibits RANKL-induced osteoclastogenesis. **(A)** Chemical structure of Sec-O-glucosylhamaudol (SOG). **(B)** Cell viability of BMMs cultured with M-CSF, RANKL, and indicated concentrations of SOG, as determined by the MTT assay. **(C)** TRAP staining of BMMs cultured for 4 d with M-CSF, RANKL, and different concentrations of SOG. **(D)** TRAP staining of RANKL-stimulated BMMs treated with 200 μM SOG for one day at different stages of cell culture. Magnification: 100×, Scale bar: 190 μm. TRAP^+^ multinucleated cells with ≥3 nuclei were regarded as osteoclasts. **(E)** SOG treatment time for **(D)**. All experiments were performed in duplicate at least three times, *p < 0.05, **p < 0.01 and ***p < 0.001 versus RANKL-positive group.

Sec-O-glucosylhamaudol (SOG) (**Figure 1A**), a flavonoid compound derived from the root of *Saposhnikovia divaricata* (Turcz. ex Ledeb.) Schischk., was reported to exhibit analgesic, anti-inflammatory, and high 5-lipoxygenase (5-LO) inhibitory effects (10–13). However, it is unclear whether it affects RANKL-induced osteoclastogenesis. This study investigated the effects of SOG on RANKL-induced osteoclastogenesis and aimed to clarify the underlying mechanisms. The potential protective effects of SOG on bone destruction in a lipopolysaccharide (LPS)-induced mouse model of bone loss were also evaluated.

## Materials and Methods

### Materials

Sec-O-glucosylhamaudol (purity ≥95%) was obtained from Nature Standard (Shanghai, China). Alpha modification of Eagle’s minimum essential medium (α-MEM), fetal bovine serum (FBS), and penicillin and streptomycin (PS) were purchased from Gibco (Grand Island, NY, USA). Recombinant murine M-CSF and RANKL were from Prospec (Rehovot, Israel). SB415286 was purchased from Selleckchem (Houston, TX, USA). LPS from *Escherichia coli* O55:B5, 2,5-diphenyltetrazolium bromide (MTT), zoledronic acid (ZOL), tartrate-resistant acid phosphatase (TRAP) staining kit, rhodamine phalloidin, 4′,6-diamidine-2′-phenylindole dihydrochloride (DAPI), and all other chemicals were obtained from Sigma-Aldrich (St Louis, MO, USA). Antibodies against phospho-P65, NFATc1, c-Fos, and PP2B-Aα were from Santa Cruz Biotechnology (Santa Cruz, CA, USA). All other antibodies were from Cell Signaling Technology (Danvers, MA, USA).

### Cell Culture and Osteoclast Differentiation

Murine bone marrow cells were separated from the femur and tibia of 6–8-week-old mice (SLAC Laboratory Animal Co., Shanghai, China) and cultured in α-MEM supplemented with 10% FBS, 1% PS, and 30 ng/mL M-CSF for 24 h as previously described (14). Adherent cells were removed and floating cells were collected by centrifuging at 1000 × *g* for 5min, re-suspending, and culturing in 10-cm dishes in α-MEM complete medium with 30 ng/mL M-CSF at 37°C, 5% CO_2_ for 2 days (d). Adherent cells were regarded as bone marrow macrophages (BMMs) and used as osteoclast precursors.

For osteoclast differentiation, BMMs (7 × 10^4^ cells/well) were cultured in 48-well plates with α-MEM complete medium supplemented with 30 ng/mL M-CSF and 100 ng/mL RANKL for 4 d. Various concentrations of SOG were added to cultures at indicated times. The TRAP staining assay was performed as described previously (14), and TRAP-positive cells containing three or more nuclei were regarded as osteoclasts.

### Cytotoxicity Assay

BMMs (1.5 × 10^4^ cells/well) were cultured in 96-well plates with α-MEM complete medium containing 30 ng/mL M-CSF, 100 ng/mL RANKL, and SOG (0–200 μM) for 24 h or 48 h. After removing the medium, 100 μL of MTT (0.5 mg/mL) solution was added to each well. After 4 h, the supernatant was discarded and 150 μL dimethyl sulfoxide was added. The optical density at 570 nm was measured using a microplate reader.

### F-Actin Ring Formation Assay

BMMs (7 × 10^4^ cells/well) were cultured in 48-well plates with α-MEM complete medium containing 30 ng/mL M-CSF, 100 ng/mL RANKL, and SOG (0–200 μM) for 4 d. Cells were fixed with 4% paraformaldehyde (PFA) for 20 min, and permeabilized with 0.5% Triton X-100 for 5 min. After washing three times with phosphate-buffered saline (PBS), phalloidin was used to stain F-actin for 30 min and DAPI was used to stain nuclei for 5 min. Fluorescent images were acquired on a fluorescence microscope (RVL-110-G; ECHO, San Diego, CA, USA).

### Mineral Resorption Assay

BMMs (1 × 10^5^ cell/well) were cultured in a Corning osteo assay surface 24-well plate with α-MEM complete medium containing 30 ng/mL M-CSF, 100 ng/mL RANKL, and SOG (0–200 μM) for 4 d. Cells were then removed with 5% sodium hypochlorite, and modified von Kossa staining was performed as previously described (15). The resorption area was observed under a light microscope and analyzed using ImageJ software (NIH, Bethesda, MD, USA). The results were expressed as a percentage of the total well area.

### Quantitative Real-Time PCR Assay

BMMs (1 × 10^6^ cells/well) were cultured in 6-well plates with α-MEM complete medium containing 30 ng/mL M-CSF, 100 ng/mL RANKL, and 200 μM SOG for the indicated periods. Total RNA was extracted using the RNA EASY MINI KIT (Qiagen, Hilden, Germany) according to the manufacturer’s instruction. The Transcriptor First Strand cDNA Synthesis Kit (Roche, Mannheim, Germany) was used to reverse-transcribe cDNA. mRNA expression levels of osteoclast-related genes were evaluated by real-time PCR using a SYBR Green kit (Roche), and glyceraldehyde 3-phosphate dehydrogenase as a control gene. PCR conditions were: 95°C for 10 min, followed by 40 cycles at 95°C for 10 s, 60°C for 15 s, and 72°C for 20 s. Primer sequences are shown in **Table 1**.

**Table 1:** Primers used for quantitative PCR (5′–3′ sequence).

Gene	Forward	Reverse
TRAP	CACTCCCACCCTGAGATTTGT	CATCGTCTGCACGGTTCTG
CTSK	AATACCTCCCTCTCGATCCTACA	TGGTTCTTGACTGGAGTAACGTA
DC-STAMP	TACGTGGAGAGAAGCAAGGAA	ACACTGAGACGTGGTTTAGGAAT
c-Fos	CGGGTTTCAACGCCGACTA	TTGGCACTAGAGACGGACAGA
NFATc1	GGAGAGTCCGAGAATCGAGAT	GGAGAGTCCGAGAATCGAGAT
GAPDH	AGGTCGGTGTGAACGGATTTG	TGTAGACCATGTAGTTGAGGTCA

### Small Interfering (si)RNA Transfection of Cells

BMMs cultured in indicated plates at 37°C for 24 h were transfected with control siRNA or 5-LO siRNA at a final concentration of 50 nM using Ribo FECTTM CP Reagent (RiboBio, Guangzhou, China) according to the manufacturer’s protocol. Control siRNA was used as a negative control for the possible nonspecific effects of RNA interference. After removing transfection mixtures, cells were cultured with α-MEM complete medium containing 30 ng/mL M-CSF and 100 ng/mL RANKL for the indicated time. TRAP staining or western blotting assays were then performed.

### Western Blotting Assays

BMMs were treated with 200 μM SOG in the presence or absence of 30 ng/mL M-CSF and 100 ng/mL RANKL for the indicated time. Cells were then collected and lysed using radioimmunoprecipitation assay lysis buffer on ice. Equal amounts of protein sample (30–50 μg) were separated by 10%–12% sodium dodecyl sulfate–polyacrylamide gel electrophoresis and transferred to polyvinylidene fluoride membranes. Membranes were blocked with 5% fat-free milk in Tris-buffered saline and Tween 20 (TBST) for 2 h and incubated with primary antibodies against target genes overnight at 4°C. After washing three times with TBST, the membranes were incubated with horseradish peroxidase-conjugated secondary antibody for 2 h at room temperature. After washing five times with TBST, the membranes were detected using the MicroChemi Chemiluminescence system (DNR Bio Imaging System, Jerusalem, Israel), and band intensities were quantified using ImageJ software.

### LPS-Induced Bone Loss Model

Male C57BL/6 mice (6-weeks-old) were obtained from Shanghai SLAC Laboratory Animal Co., Ltd. (Shanghai, China). Animal experiments were performed strictly according to the Guide for the Humane Use and Care of Laboratory Animals (Animal protocol number: TJCAC-018-036). Mice were randomly assigned to five groups (n=10 per group): PBS control (Sham group), LPS injection (LPS group; 5 mg/kg body weight), SOG injection (only SOG group, 10 mg/kg body weight), LPS with 5 mg/kg body weight dose of zoledronic acid (ZOL group), and LPS with 10 mg/kg body weight dose of SOG (SOG group). SOG, ZOL, and LPS were injected intraperitoneally every other day for 8 d. A total of 2 h after SOG or ZOL injection, LPS or PBS were injected intraperitoneally. Mice were euthanized on d 8 after the first injection of LPS. Left and right femurs were collected for micro-computed tomography (CT) and histomorphometric analysis, respectively.

### Micro-CT and Histomorphometric Analysis

Left femurs were fixed with 4% PFA for 24 h, and scanned by micro-CT using a Quantum GX microCT system (PerkinElmer, Waltham, MA, USA). The bone mineral density (BMD), bone volume/total volume (BV/TV), and trabecular number (Tb.N), trabecular thickness (Tb.th) and decreased trabecular separation/spacing (Tb.Sp) were measured.

Right femurs were demineralized in 10% ethylenediaminetetraacetic acid for 2 weeks. They were then embedded in paraffin, cut into 5 μm-thick sections, and stained with H&E and TRAP. Staining was visualized using a high-quality microscope (RVL-110-G; ECHO, San Diego, CA, USA).

### Statistical Analysis

GraphPad Prism 7 software (San Diego, CA, USA) was used for data analysis. Data were expressed as means ± SD. Differences between groups were analyzed using one-way analysis of variance followed by the Student–Newman–Keuls test. *P* < 0.05 was considered statistically significant.

## Results

### SOG Inhibits RANKL-Induced Osteoclast Formation

To determine whether SOG influences RANKL-mediated osteoclast differentiation, BMMs were cultured in the presence of M-CSF, RANKL, and indicated concentrations of SOG. SOG showed no obvious cytotoxicity at a concentration range of 50–200 μM (**Figure 1B**). As shown in **Figure 1C**, RANKL significantly induced the formation of TRAP-positive multinucleated cells, which was inhibited by SOG treatment in a concentration-dependent manner.

Next, the effects of SOG on RANKL-induced osteoclastogenesis during different stages of differentiation were investigated. SOG (200 μM) treatment significantly reduced the number and size of osteoclasts at all stages of cell differentiation, with stronger inhibitory effects on osteoclast formation seen during middle and late stages (d 3 and d 4) compared with early stages of cell differentiation (d 1 and d 2) (**Figures 1D, E**). Taken together, these results suggest that SOG suppresses RANKL-induced osteoclastogenesis, especially in the middle–late stages of osteoclast differentiation.

### SOG Impairs the Formation of Resorption Pits and F-Actin Rings by Osteoclasts

**Figure 2:**
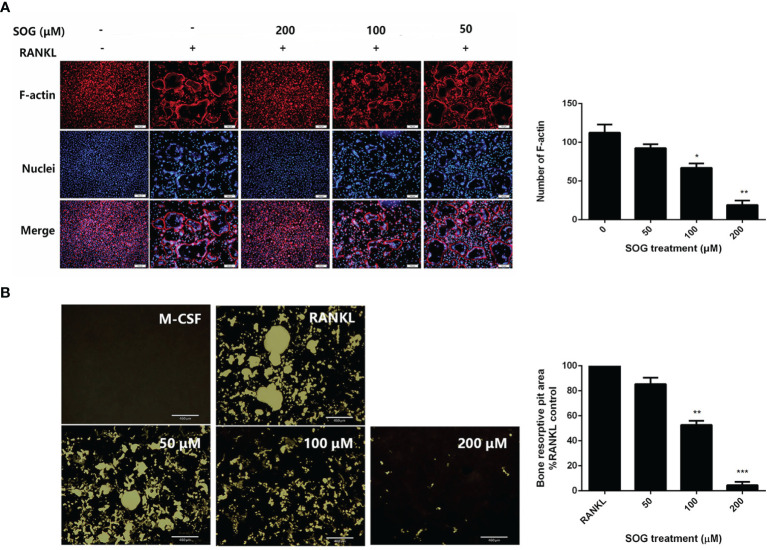
SOG repressed the formation of F-actin ring resorption pits in RANKL-stimulated BMMs. **(A)** F-actin ring staining of BMMs cultured for 4 d with M-CSF, RANKL, and different concentrations of SOG. 100× magnification. Scale bars, 100 μm. **(B)** Von Kossa staining of BMMs cultured for 4 d with M-CSF, RANKL, and different concentrations of SOG. Resorption pits were observed under a light microscope. 40× magnification. Scale bars, 460 μm. All experiments were performed in duplicate at least three times. *p < 0.05, **p < 0.01, ***p < 0.001 versus RANKL-positive group.

Previous studies reported that in osteoclasts plated on bone slices, dynamin was enriched in the actin-rich sealing zone that delineates the resorptive space (16). Similarly, when plated on serum-coated coverslips or regular plates, dynamin was highly concentrated at the periphery of the cell, where podosomes are localized as a belt, this belt is called F-actin ring. The formation of a well-defined F-actin ring is known to be essential for the bone resorption function of osteoclasts (4). Therefore, we examined the effects of SOG treatment on RANKL-induced osteoclast F-actin ring formation. **Figure 2A** shows that the number and size of F-actin rings were significantly reduced with increasing concentrations of SOG treatment of RANKL-stimulated BMMs. SOG treatment also significantly dose-dependently decreased the formation of resorption pits, as shown in **Figure 2B**. At a concentration of 200 μM, SOG treatment almost completely abolished the formation of F-actin rings and resorption pits in RANKL-stimulated BMMs. Meanwhile, 200 μM SOG was added to BMMs at day 1, 2, 3 and 4 during osteoclastogenesis for 24 h. SOG exhibited stronger inhibition on bone resorption pits formation at middle-late stage (day 3 and day 4 after RANKL treatment) compared to that at early stage (**Figure S1**). These results suggest that SOG inhibits RANKL-induced mineral resorption and formation of F-actin rings through inhibition of RANKL-induced osteoclast formation.

### SOG Represses the Induction of Osteoclast-Specific Gene Expression by RANKL

**Figure 3:**
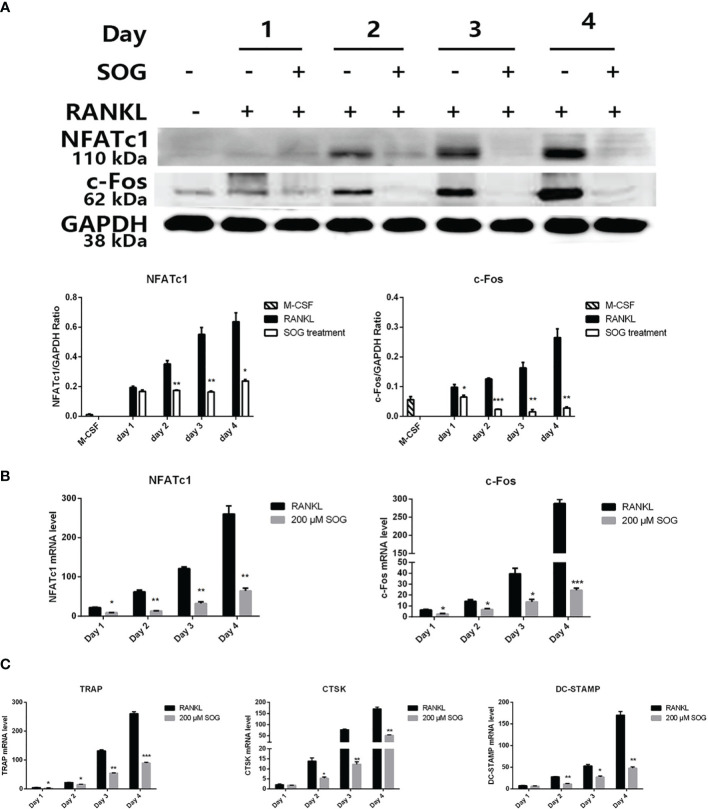
SOG reduced the RANKL-induced expression of c-Fos, NFATc1, and osteoclast-specific genes in BMMs cultured with M-CSF, RANKL, and 200 μM SOG for up to 4 d **(A)** c-Fos and NFATc1 protein levels as determined by western blotting analysis. Real-time PCR analysis of *c-Fos* and *NFATc1* **(B)** and *TRAP*, *CTSK*, and *DC-STAMP* **(C)** expression. All experiments were performed at least three times. *p < 0.05, **p < 0.01, and ***p < 0.001 versus RANKL-positive group.

RANKL stimulation up-regulates osteoclast-related genes, which control the differentiation, maturation, and function of osteoclasts, while master transcription factor NFATc1 regulates the expression of osteoclast-specific genes (17). We therefore investigated the effects of SOG on the expression of NFATc1 and c-Fos, and several osteoclast marker genes including *CTSK*, *TRAP*, and *DC-STAMP*. SOG treatment significantly suppressed the induction of c-FOS and NFATc1 by RANKL at both the mRNA and protein level, in a time-dependent manner (**Figures 3A, B**). **Figure 3C** also shows that SOG treatment significantly suppressed mRNA expression levels of *CTSK*, *TRAP*, and *DC-STAMP* in a time-dependent manner in RANKL-stimulated BMMs. These results suggest that SOG inhibits osteoclastogenesis by suppressing the expression of c-Fos/NFATc1-mediated osteoclast-specific genes.

### The Effects of SOG on NF-κB, MAPK, and AKT-GSK3β Pathways During the Initial Stage of Osteoclastogenesis

**Figure 4:**
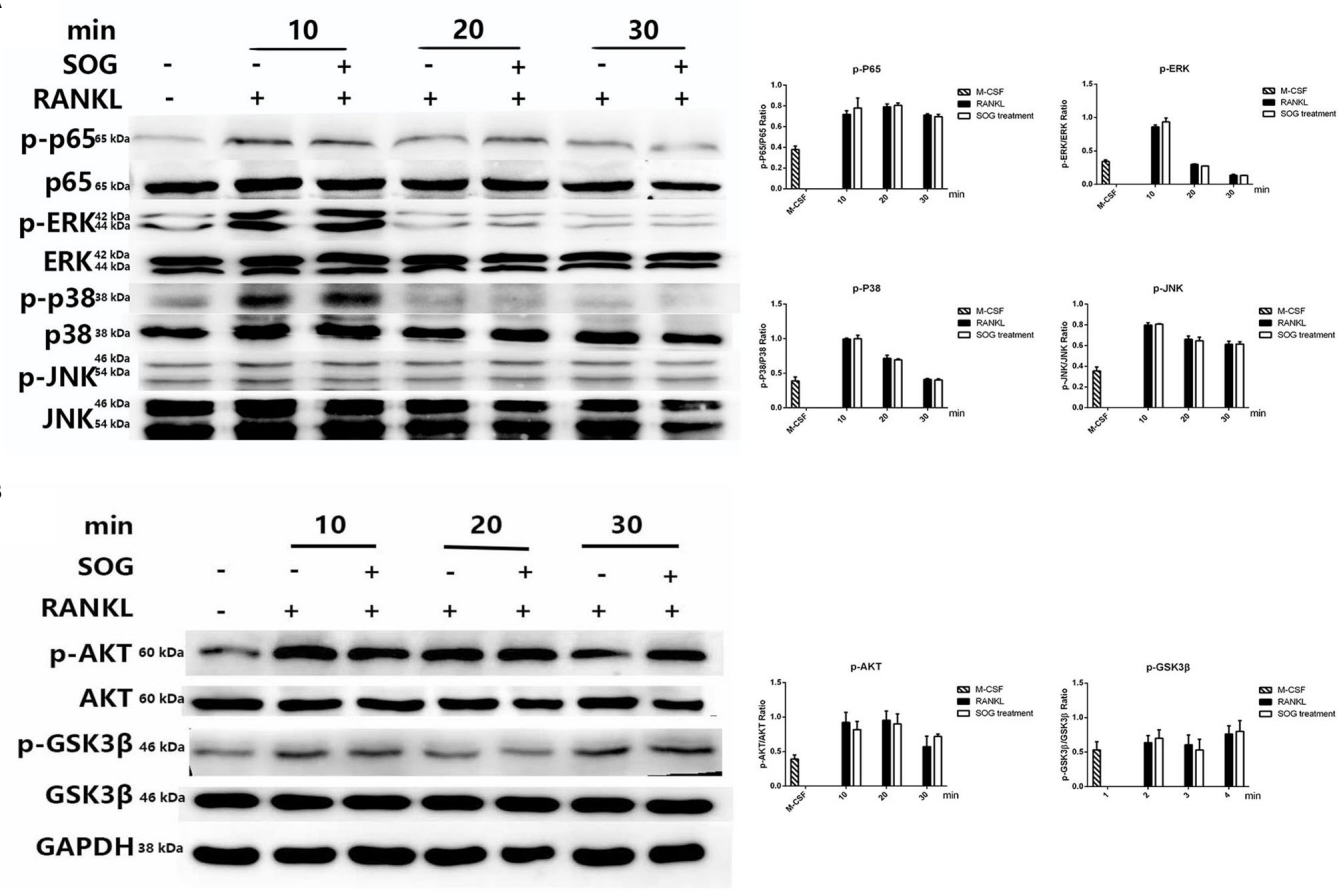
The effects of 200 µM SOG pretreatment on 100 ng/mL RANKL-induced NF-κB, MAPK, and AKT-GSK3β signaling in BMMs as assessed by western blotting. **(A)** Phosphorylation of the NF-κB p65 subunit and MAPKs including ERK1/2, JNK, and p65. **(B)** Phosphorylation of AKT and GSK3β. Relative band intensities of phosphorylated proteins were normalized to that of the un-phosphorylated protein. All experiments were performed at least three times.

NF-κB, MAPK, and AKT-GSK3β pathways play a vital role in the early induction of NFATc1 (18–20). To further explore the molecular mechanism by which SOG modulates NFATc1 signaling, we evaluated its effects on the RANKL-initiated transient activation of NF-κB, MAPK, and AKT, and the inactivation of GSK3β using western blotting. As shown in **Figure 4A**, RANKL stimulation induced the transient phosphorylation of the NF-κB p65 subunit and MAPKs including p38, extracellular signal−regulated kinase (ERK)1/2, and c-Jun N-terminal kinase (JNK), which was unchanged by SOG treatment. The RANKL-induced transient phosphorylation of AKT and GSK3β was also unchanged by SOG treatment (**Figure 4B**). These results indicate that SOG does not alter the RANKL-induced transient activation of NF-κB, MAPK, and AKT signaling, implying that it might not influence the initial induction of NFATc1 during the early stages of osteoclast differentiation.

### The Effects of SOG on RANKL-Induced Calcineurin Expression and the AKT/GSK3β Signaling Pathway During Osteoclastogenesis

**Figure 5:**
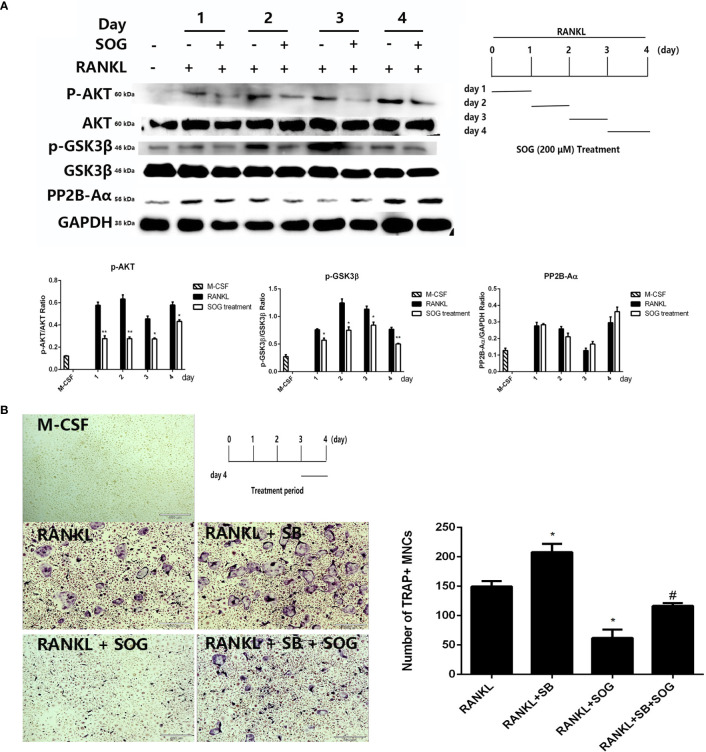
The effect of SOG on RANKL-induced calcineurin expression and the AKT-GSK3β signaling pathway. **(A)** Protein levels of calcimeurin, AKT, and GSK3β in BMMs at different periods of osteoclastogenesis cultured with M-CSF, RANKL, and SOG, as determined by western blotting (left panel). The SOG treatment time is shown in the lower right panel. **(B)** TRAP staining of BMMs cultured with M-CSF, RANKL, and SOG or SB415286 (SB) at d 3 of osteoclastogenesis (left panel). The SOG treatment time is shown in the upper left panel. TRAP^+^ cells with ≥3 nuclei were counted (right panel). 40× magnification. Scale bars, 460 μm. All experiments were performed in duplicate at least three times. *p < 0.05 and **p < 0.01, versus RANKL-induced group. ^#^*p* < 0.05 versus RANKL + SB-induced group.

NFATc1 activation is regulated by calcineurin-mediated NFATc1 dephosphorylation and GSK3β-mediated NFATc1 phosphorylation during the middle–late stage of osteoclastogenesis (21). To investigate whether SOG affects NFATc1 activity at the middle–late stage of cell differentiation, the phosphorylation of AKT and GSK3β and protein levels of the calcineurin catalytic subunit PP2B-Aα were detected during different stages of osteoclastogenesis. RANKL-stimulated BMMs were treated with 200 µM SOG for 24 h at different periods of osteoclastogenesis. As shown in **Figure 5A**, SOG treatment had no effect on the protein levels of the calcineurin catalytic subunit PP2B-Aα in RANKL-stimulated BMMs. However, the phosphorylation of AKT and GSK3β was clearly inhibited by SOG in a time-dependent manner in RANKL-stimulated BMMs during the middle–late stage of osteoclast differentiation. These results suggest that SOG suppresses NFATc1 activation by inhibiting AKT-mediated GSK3β inactivation.

Further, the selective GSK3β inhibitor SB415286 was used to determine whether SOG suppresses RANKL-induced osteoclast differentiation through blocking AKT-mediated GSK3β inactivation. As shown in **Figure 5B**, the addition of 10 μM SB415286 weakened the inhibitory effect of SOG on osteoclast formation during the middle–late stage of cell differentiation. Taken together, these data suggest that SOG represses RANKL-induced osteoclast differentiation at least in part by inhibiting AKT-mediated GSK3β inactivation during the middle–late stage of osteoclastogenesis.

### The Effects of 5-LO Knockdown on AKT-Mediated GSK3β Inactivation

**Figure 6:**
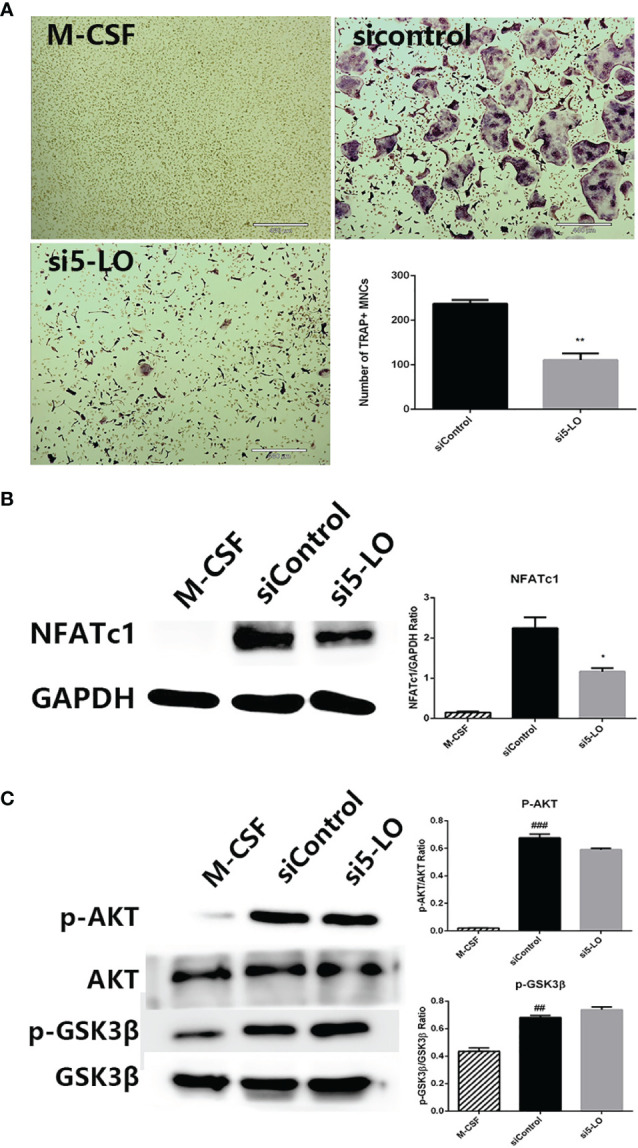
The effects of 5-LO knockdown on RANKL-induced osteoclastogenesis. **(A)** TRAP staining of BMMs transfected with control siRNA or 5-LO targeted siRNA and cultured with M-CSF and RANKL for 4 d. 40× magnification. Scale bars, 460 μm. **(B)** Western blotting of NFATc1 and GAPDH protein expression in cells treated as in **(A)**. Relative protein expression was quantified using ImageJ software (right panel). **(C)** Western blotting of AKT, and GSK3β protein expression in cells treated as in **(A)**. Relative protein expression was quantified using ImageJ software (right panel). All experiments were performed in duplicate at least three times. ^##^*p* < 0.01 and ^###^*p* < 0.001 versus M-CSF group. *p < 0.05 and **p < 0.01 versus siControl group.

SOG was previously reported to inhibit 5-LO catalytic activity *in vitro* with an IC_50_ value of 7.45 μM (13). To explore whether SOG blocks AKT-mediated GSK3β inactivation by inhibiting 5-LO, we silenced the expression of 5-LO in BMMs by 5-LO targeted siRNA transfection. As shown in **Figure 6A**, 5-LO knockdown significantly inhibited osteoclast formation compared with the control group. Additionally, NFATc1 induction by RANKL was significantly attenuated in 5-LO siRNA-transfected BMMs compared with controls (**Figure 6B**). However, 5-LO knockdown did not change the phosphorylation levels of AKT or GSK3β (**Figure 6C**). Taken together, these data indicate that 5-LO knockdown suppresses RANKL-induced osteoclastogenesis, but not through the AKT/GSK3β signaling pathway. SOG might also suppress RANKL-induced osteoclastogenesis by inhibiting both the independent 5-LO pathway and AKT-mediated GSK3β inactivation.

### SOG Treatment Improves LPS-Induced Bone Loss in Mice

**Figure 7:**
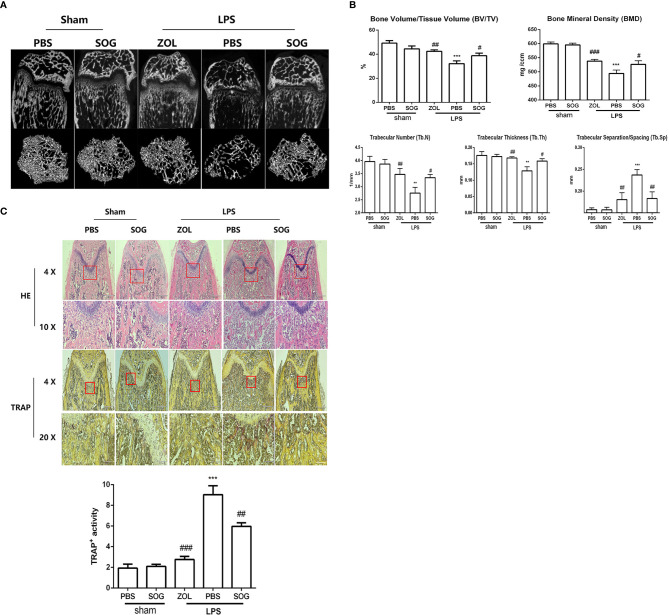
SOG improved LPS-induced bone loss in mice. **(A)** Representative 2D and 3D reconstructions of micro-CT scans of femurs. **(B)** Bone volume/Tissue Volume (BV/TV), Bone mineral density (BMD), trabecular number (Tb.N), trabecular separation/spacing (Tb.Sp), and trabecular thickness (Tb.Th) of femurs were quantified by micro-CT data analysis using CT analyzer software. **(C)** Histological sections of femoral bone tissue sections stained with H&E (at 40× and 100× magnification), TRAP staining (at 40× and 200× magnification) and quantification of TRAP staining. Data are expressed as means ± SD. *p < 0.05, **p < 0.01, and ***p < 0.001 versus sham group; ^#^*p* < 0.05, ^##^*p* < 0.01, and ^###^*p* < 0.001 versus LPS group.

Given the inhibitory effects of SOG on osteoclastogenesis *in vitro*, we next evaluated the potential therapeutic effects of SOG in an LPS-induced mouse model of bone loss. Two- and three-dimensional reconstruction images of micro-CT revealed that the LPS group exhibited significant femur destruction compared with the sham group (**Figure 7A**). However, SOG treatment significantly improved bone destruction in LPS-treated mice. Similarly, zoledronic acid (ZOL), a known anti-bone resorptive drug used as a positive compound, markedly reduced LPS-induced bone loss. Quantitative analysis of bone microparameters showed that SOG treatment increased the values of bone mineral density (BMD), bone volume/total volume (BV/TV), and trabecular number (Tb.N), trabecular thickness (Tb.th) and decreased trabecular separation/spacing (Tb.Sp) compared with the LPS-treated group (**Figure 7B**). Hematoxylin and eosin (H&E) staining showed that SOG treatment significantly reduced LPS-induced bone destruction (**Figure 7C**). Moreover, TRAP staining revealed that SOG decreased the number of TRAP-positive osteoclasts compared with the LPS group (**Figure 7C**). These observations suggest that SOG attenuates LPS-induced bone loss in mice.

## Discussion

In this study, we demonstrated for the first time that SOG significantly suppresses RANKL-induced osteoclastogenesis *in vitro*, and that it markedly improves LPS-induced bone loss in a mouse model. Thus, SOG has the potential to be a new treatment option for osteoclast-related bone lysis diseases.

Osteoclastogenesis is a complicated disease involving multiple stages. We explored the effects of SOG on osteoclast differentiation at different stages of cell differentiation, and found that it exhibited inhibitory effects during all periods of osteoclastogenesis, but had stronger inhibitory effects on osteoclast formation in the middle–late stage of cell differentiation.

NFATc1 is known to play a crucial role in RANKL-induced osteoclastogenesis by inducing the expression of osteoclast-specific genes such as *TRAP*, *CTSK*, and *DC-STAMP* (22). *CTSK* encodes a lysosomal cysteine proteinase involved in bone remodeling and resorption (23, 24), while *DC-STAMP* functions in osteoclast fusion and maturation (25, 26). This study demonstrated that SOG significantly suppressed the induction of key transcription factors NFATc1 and c-Fos at both mRNA and protein levels. Moreover, SOG also significantly inhibited the transcription of *TRAP*, *CTSK*, and *DC-STAMP*. This shows that SOG suppresses osteoclastogenesis by inhibiting NFATc1 and c-Fos and their target genes.

The association of RANKL with its receptor RANK initiates early signaling pathways such as NF-κB, MAPK, and AKT signaling (27–31), which induce the expression of NFATc1. We observed no inhibitory effects of SOG on phosphorylation of the NF-κB p65 subunit, MAPKs (p38, JNK, and ERK1/2), AKT, or GSK3β after RANKL stimulation. These findings suggest that SOG might not influence the initial induction of NFATc1 by modulating signaling pathways in the early stage of osteoclastogenesis.

NFATc1 maintains its auto-amplification by activating the transcription of NFATc1 (22). Calcineurin mediates NFATc1 translocation into the nucleus and induces the expression of its target genes, including itself, through dephosphorylating NFATc1 at its serine residues (32). The calcineurin inhibitor FK506 was reported to inhibit RANKL-induced osteoclastogenesis, even when it was added at a late phase of osteoclastogenesis (22). However, we showed that SOG did not alter expression of the calcineurin catalytic subunit PP2B-Aα, indicating that it might not affect NFATc1 dephosphorylation by inhibiting calcineurin.

GSK3β-mediated phosphorylation of NFATc1 at serine residues promotes NFATc1 nuclear export and localization in the cytoplasm (33, 34). RANKL stimulates the phosphorylation of GSK3β at serine 9, leading to its inactivation, the blockage of NFATc1 phosphorylation, and promotion of NFATc1 nuclear localization. The phosphorylation of GSK3β at serine 9 is a continuous process that peaks on d 3 after RANKL stimulation (35). Accordingly, we showed that the relative level of GSK3β phosphorylation reached its peak on d 3 of RANKL stimulation. The ectopic expression of a constitutively active form of GSK-3β was previously found to attenuate osteoclast formation by downregulating NFATc1 (36). Consistent with this, we showed that RANKL-induced GSK3β inactivation was markedly reduced by SOG treatment at different stages of osteoclastogenesis. Even the addition of SOG at the middle–late phase of osteoclastogenesis (d 3 and d 4 after RANKL stimulation) still remarkably suppressed GSK3β phosphorylation. These results suggest that SOG promotes NFATc1 phosphorylation through enhancing GSK3β activation.

The GSK3β inhibitors kenpaullone, SB216763, and SB415286 previously promoted RANKL-induced osteoclastogenesis (35). In this study, we found that the addition of 10 μM SB415286 slightly weakened the inhibitory effect of SOG on osteoclast formation in the middle–late stage of cell differentiation, suggesting that SOG inhibits osteoclast formation at least in part through activating GSK3β.

Previous studies reported a crucial role for AKT/GSK3β signaling in osteoclastogenesis (36). RANKL-activated AKT enhanced GSK3β phosphorylation at serine 9, causing its inactivation, increasing NFATc1 accumulation in the nucleus, and promoting osteoclastogenesis (37). Moreover, the ectopic expression of constitutively activated AKT increased inactive GSK3β levels, thereby enhancing the nuclear localization of NFATc1 (36). Consistent with this, we found that SOG strongly inhibited RANKL-induced AKT phosphorylation in the middle–late stage of osteoclastogenesis (d 2–d 4). In addition, our study also found that SOG did not inhibit RANKL-induced PI3K activation (**Figure S2**). It is well known that PI3K directly phosphorylates AKT on T308 and S473 (38). Besides of direct activation of PI3K on AKT, SH2-containing inositol phosphatase (SHIP) and phosphatase and tensin homolog (PTEN), both negative regulators of PI3K, inhibit AKT phosphorylation by PI3K (39, 40). Our results demonstrated that SOG did not alter the protein levels of PTEN (**Figure S2**). Recent studies reported that IKKα and mTOR complex 2 (mTORC2) directly phosphorylate AKT (41). These AKT regulator might be potential target of SOG. Taken together, these results suggest that SOG suppresses osteoclast formation, at least in part, by inhibiting the AKT/GSK3β/NFATc1 signaling pathway.

Leukotrienes are inflammatory mediators that play an important role in the development of inflammation. Several studies reported that osteoclastogenesis was promoted by leukotriene B4 *via* G protein-coupled receptors BLT1 and BLT2, and by cysteinyl leukotrienes *via* G protein-coupled CysLT receptors (42–45). 5-LO is a key enzyme of the arachidonic acid cascade that catalyzes the formation of bioactive leukotrienes, and plays an important role in inflammatory disorders and bone metabolism (46). Lee et al. silenced 5-LO expression to diminish RANKL-induced osteoclastogenesis, showing that the inhibitory effects of 5-LO knockdown on osteoclastogenesis might be contributed to the blockage of leukotriene autocrine signaling (45). Another study found that the 5-LO inhibitor K7 markedly suppressed osteoclastogenesis by reducing NFATc1 expression (47), while SOG exhibited an inhibitory potency on 5-LO catalytic activity with an IC_50_ value of 7.45 μM *in vitro* (13). In a latest study, Liu et al. reported that SOG causes the conformational and micro-environmental changes of 5-LO *via* direct binding with 5-LO with the hydrogen bonding and electrostatic force (48). Consistent with this, we showed the effective suppression of RANKL-induced osteoclast formation at a SOG concentration of 50–200 μM. Thus, we speculate that SOG also suppresses RANKL-induced osteoclastogenesis by inhibiting 5-LO activity and blocking the production of leukotrienes. 5-LO expression silencing by targeted siRNA in our study resulted in a reduction of NFATc1 expression, without altering the phosphorylation of AKT or GSK3β. This provided further support for SOG suppression of RANKL-induced osteoclastogenesis through inhibiting the independent 5-LO pathway, as well as AKT-mediated GSK3β inactivation.

LPS-induced osteolytic model was widely used in inflammatory bone loss diseases caused by osteoclast hyperactivation (49, 50). In this study, we established an LPS-induced bone loss mouse model to investigate whether SOG could improve bone destruction. H&E staining and micro-CT of femurs showed that SOG effectively rescued LPS-induced bone loss, while TRAP staining showed that SOG decreased the number of osteoclasts, which is consistent with *in vitro* findings.

In conclusion, SOG attenuated the formation and function of osteoclasts by inhibiting both the independent 5-LO pathway and AKT-mediated GSK3β inactivation. Moreover, SOG prevented LPS-induced bone loss in mice through inhibiting osteoclastogenesis.

## Data Availability Statement

The original contributions presented in the study are included in the article/**Supplementary Material**. Further inquiries can be directed to the corresponding authors.

## Ethics Statement

Animal experiments were performed strictly according to the Guide for the Humane Use and Care of Laboratory Animals (Animal protocol number: TJCAC-018-036).

## Author Contributions

Conceptualization, HW, ZC, and WS. methodology, data curation, validation, JC and M-XZ. formal analysis, CW. investigation, XC. resources, software, MS. writing—original draft preparation, JC. Supervision, QP. writing—review and editing, WS. funding acquisition, HW. All authors have read and agreed to the published version of the manuscript. All authors contributed to the article and approved the submitted version.

## Funding

This research was funded by the National Natural Science Foundation (grant no. 31670347, 81001369, 31170327 and 31870121) and Shanghai Science and Technology Commission of Shanghai Municipality (No. 21015800500).

## Conflict of Interest

The authors declare that the research was conducted in the absence of any commercial or financial relationships that could be construed as a potential conflict of interest.

## Publisher’s Note

All claims expressed in this article are solely those of the authors and do not necessarily represent those of their affiliated organizations, or those of the publisher, the editors and the reviewers. Any product that may be evaluated in this article, or claim that may be made by its manufacturer, is not guaranteed or endorsed by the publisher.
