# Supplementary material for: Sec-O-Glucosylhamaudol Inhibits RANKL-Induced Osteoclastogenesis by Repressing 5-LO and AKT/GSK3β Signaling
Source: Front Immunol. 2022 Apr 26;13:880988. doi: 10.3389/fimmu.2022.880988 (PMC9087042; doi:10.3389/fimmu.2022.880988)
Supplement: Supplementary file 1 [file DataSheet_1.docx]

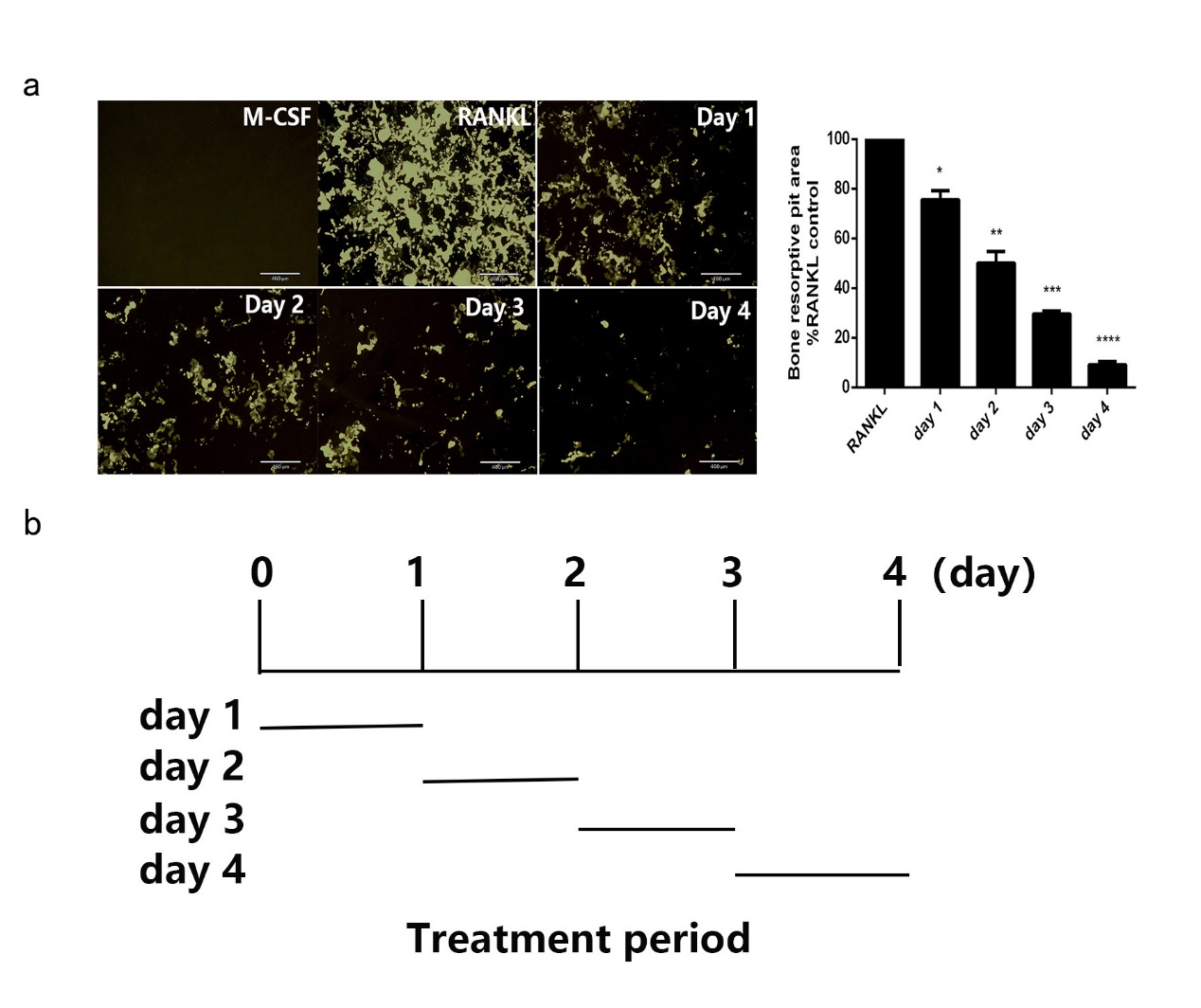


Figure S1 Effects of 200 μM SOG on RANKL-induced osteoclast differentiation from BMMs at different stage of cell differentiation. 200 μM SOG were added to culture medium at indicated time point during cell culture period. (a) The resorption pit areas were observed at a light microscope. 40× magnification. Scale bars, 460 μm. Quantification of resorption pit areas per view area (right panel). All experiments were performed in duplicate at least three times, *p < 0.05, **p < 0.01, ***p < 0.001 and ****p < 0.0001 versus RANKL induced group. (b) Treatment time of SOG.


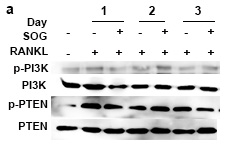


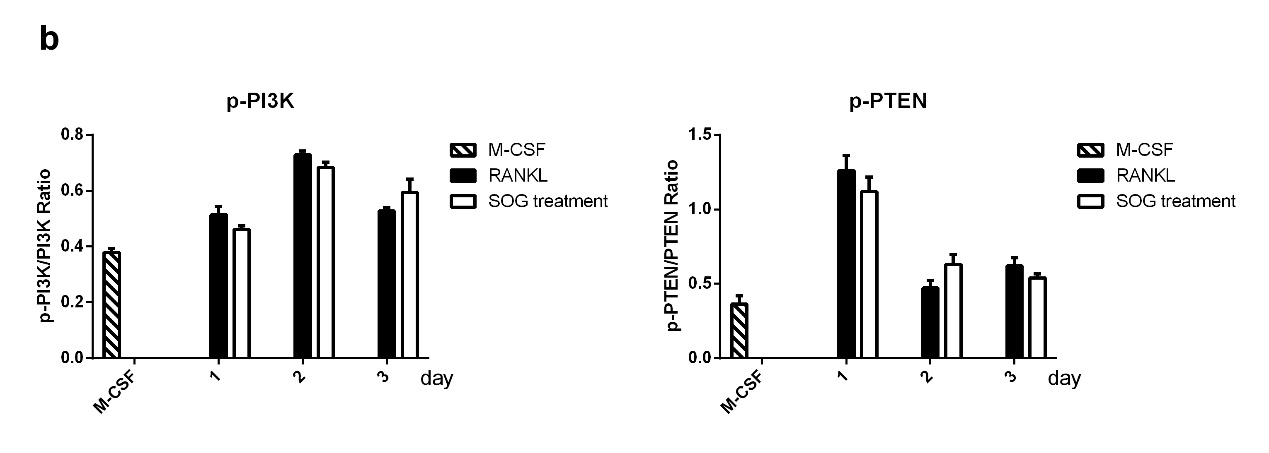


Figure S2: The effect of SOG on RANKL-induced PI3K/PTEN pathway. BMMs were cultured in α-MEM completed medium containing 30 ng/mL M-CSF, 100 ng/mL RANKL and 200 μM SOG at different periods of osteoclastogenesis. (a) The protein levels of p-PI3K, PI3K, p-PTEN and PTEN were analyzed using Western blotting with specific antibodies. (b) Relative protein expression was calculated using Image J. All experiments were performed at least three times
